# Case Report: Multiple Causes of Cardiac Death After the First Infusion of Atezolizumab: Histopathological and Immunohistochemical Findings

**DOI:** 10.3389/fimmu.2022.871542

**Published:** 2022-03-31

**Authors:** Ekaterina Kushnareva, Maria Stepanova, Elizaveta Artemeva, Tatyana Shuginova, Vladimir Kushnarev, Maria Simakova, Fedor Moiseenko, Olga Moiseeva

**Affiliations:** ^1^ Personalized Medicine Centre, Almazov National Medical Research Centre, Saint Petersburg, Russia; ^2^ Clinical Research and Practical Center for Specialized Oncological Care, Saint Petersburg, Russia; ^3^ N.N. Petrov National Medical Research Center of Oncology, Saint-Petersburg, Russia; ^4^ Noncoronary Heart Disease Department, Almazov National Medical Research Centre, Saint Petersburg, Russia

**Keywords:** atezolizumab, mucinous carcinoma of the lung, metastasis, myocarditis, myocardial infarction, cardiovascular toxicity

## Abstract

Immune checkpoint inhibitors are promising agents for anticancer therapy. But despite their high efficacy in the treatment of solid tumors, there is still a problem with immune-related adverse events, especially cardiovascular complications with a very high mortality rate. Myocarditis or ischemic heart disease progression is not the only possible cause of cardiovascular death in patients treated with checkpoint inhibitors. We report a case of a patient with mucinous carcinoma of the lung, with a previous history of hypertension and moderate left ventricular dysfunction. The patient was prescribed atezolizumab, but the first atezolizumab infusion resulted in the patient cardiovascular death. Postmortem histopathological evaluation of myocardium revealed several possible reasons for hemodynamic instability: tumor embolism of the coronary arteries, micrometastases of mucinous carcinoma in the myocardium, and myocarditis diagnosed by both Dallas and immunohistochemistry criteria. In addition, testing for expression of PD-L1 detected the high levels of membranous and cytoplasmic PD-L1 protein even in the myocardium area free from tumor cells. The present clinical case demonstrates a problem of cardiovascular death in patients treated with checkpoint inhibitors and actualizes the need for future research of potential risk factors for cardiovascular complications.

## Introduction

Immune checkpoint inhibitors (ICI) allowed to significantly prolong disease-free and overall survival in oncology patients who previously had a poor prognosis.

On the other hand, despite the impressive anticancer efficacy, the number of life-threatening immune-related adverse events (irAEs) rapidly increase (1). One of the irAEs is autoimmune myocarditis with poor survival and challenging diagnosis and management. However, the cause of cardiovascular (CV) death in ICI-treated patients might be more complex than only myocarditis or only myocardial infarction (MI).

**Figure 1:**
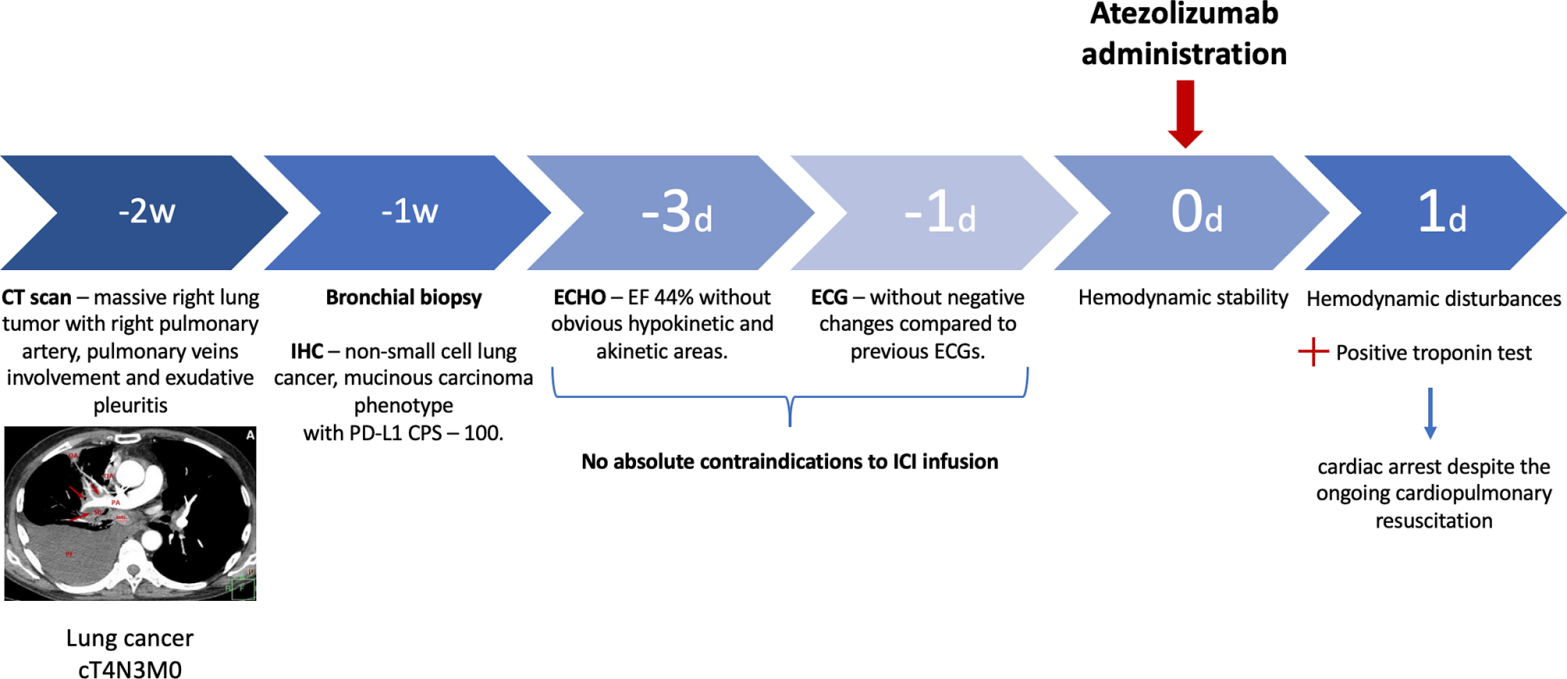
Timeline. D, day; W, week; IHC, immunohistochemistry; CPS, combined positive score; ECHO, echocardiogram; ECG, electrocardiogram; ICI, immune checkpoint inhibitor.

We present a clinical case of ICI-related fatal fulminant myocarditis in the patient after the first infusion of anti-PD-L1 ICI (**Figure 1**). Additionally, we demonstrate myocardium histopathological and immunohistochemical (IHC) evaluation with a discussion of extra findings.

## Case Report

**Figure 2:**
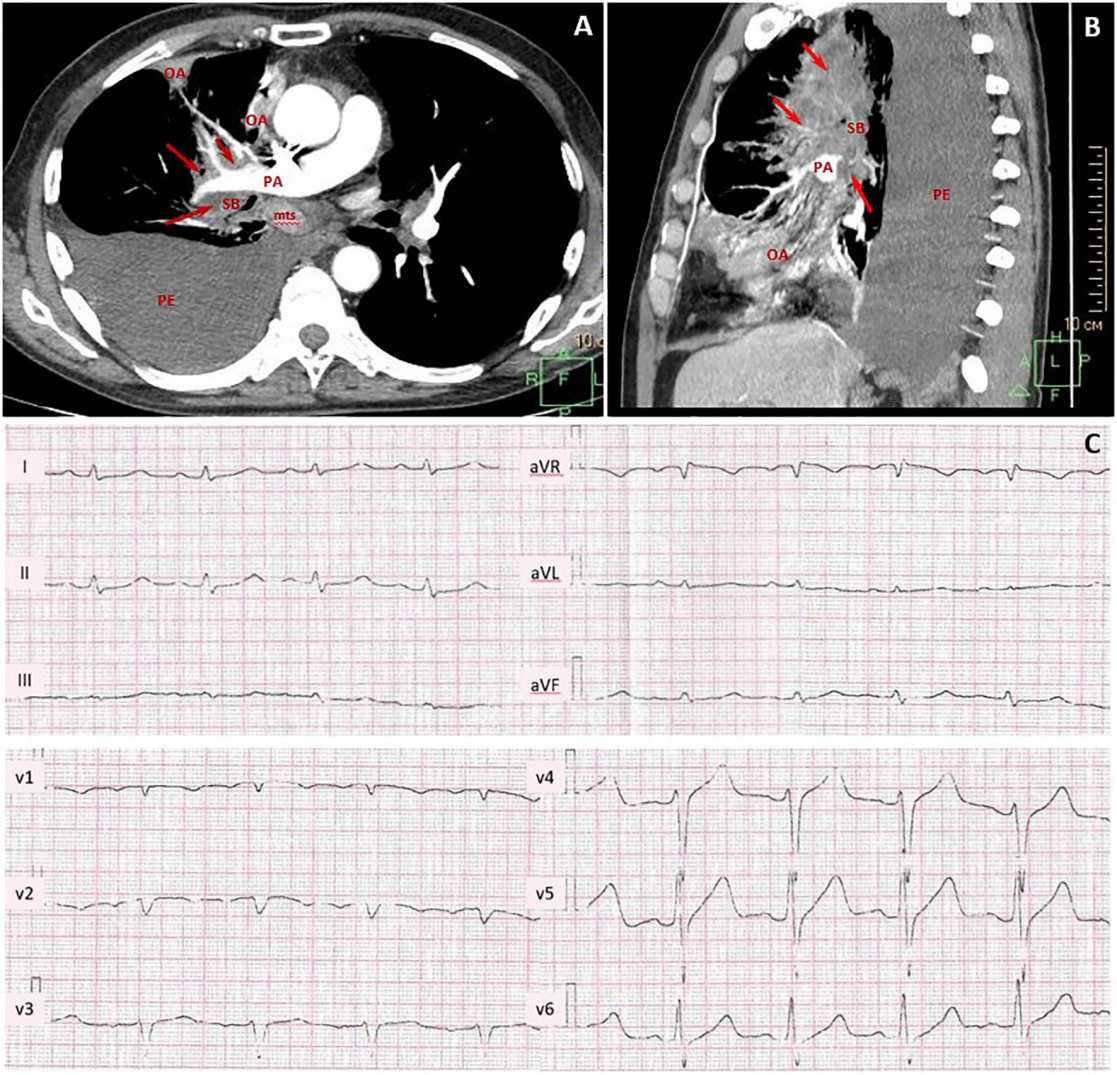
Contrast-enhanced chest CT scans and pretreatment electrocardiogram. CT maximum intensity projection reconstruction **(A)** axial plane and **(B)** sagittal plane: massive lung tumor with right pulmonary artery, pulmonary veins, lobar and segmental bronchi involvement (red arrows, tumor; PA, pulmonary artery; SB, segmental bronchus; OA, obstructive atelectasis; mts, bifurcation lymph node metastasis; PE, large right-sided pleural effusion). **(C)** Pretreatment ECG with sinus tachycardia, incomplete right bundle branch block, and poor progression of R-wave in V1–V3 leads.

A 67-year-old man was diagnosed with lung mucinous carcinoma, staged as cT4N3M0 with spreading to the mediastinum, right pulmonary artery (PA) circular involvement, and exudative pleuritis according to CT (**Figure 2A**). PD-L1 expression was in 90% of the tumor and 10% of immune cells with a combined positive score (CPS) 100. At the time of initial examination, the patient was weak and had an Eastern Cooperative Oncology Group (ECOG) performance status of 2. The patient had a long previous history of hypertension (HP) and received angiotensin-converting enzyme inhibitors and beta-blockers.

Pretreatment electrocardiogram (ECG) showed sinus tachycardia with the heart rate of 111 beats per minute, incomplete right bundle branch block, and poor R-wave progression in V1–V3 leads without negative changes as compared to previous ECGs (**Figure 2B**). Echocardiogram (ECHO) demonstrated moderate left ventricle (LV) dysfunction (ejection fraction (EF) by Simpson was 44%) without obvious hypokinetic and akinetic areas. The patient had a Khorana score of 2 points, corresponding to “intermediate thrombotic risk”; therefore, thromboprophylaxis with low-molecular-weight heparin was started.

Given the ECOG-2, high risk of CV complications related to chemotherapy and radiation therapy, and high level of PD-L1 (CPS 100), the first-line treatment with anti-PD-1 ICI was initiated.

The next evening after the first cycle of atezolizumab, the patient developed worsening shortness of breath with a respiration rate of 28 breaths per minute. During a primary evaluation, hemodynamic disturbances, including a heart rate of 144 beats per minute and blood pressure of 60/40 mmHg, were found.

The patient was delivered to the intensive care unit. Laboratory data showed a slight increase in creatine kinase-MB level (CK-MB, 26.48 U/L) and positive qualitative troponin test with normal renal and liver function. Shortly after the first symptoms, cardiac arrest occurred despite the ongoing cardiopulmonary resuscitation.

A postmortem examination revealed that a high-density tumor was observed in the root of the lung, at the upper and middle lobes. The mass embraces the PA and lobar bronchi like a muff. Postmortem blood coagulation was detected in the PA lumen without evidence of thrombus.

**Figure 3:**
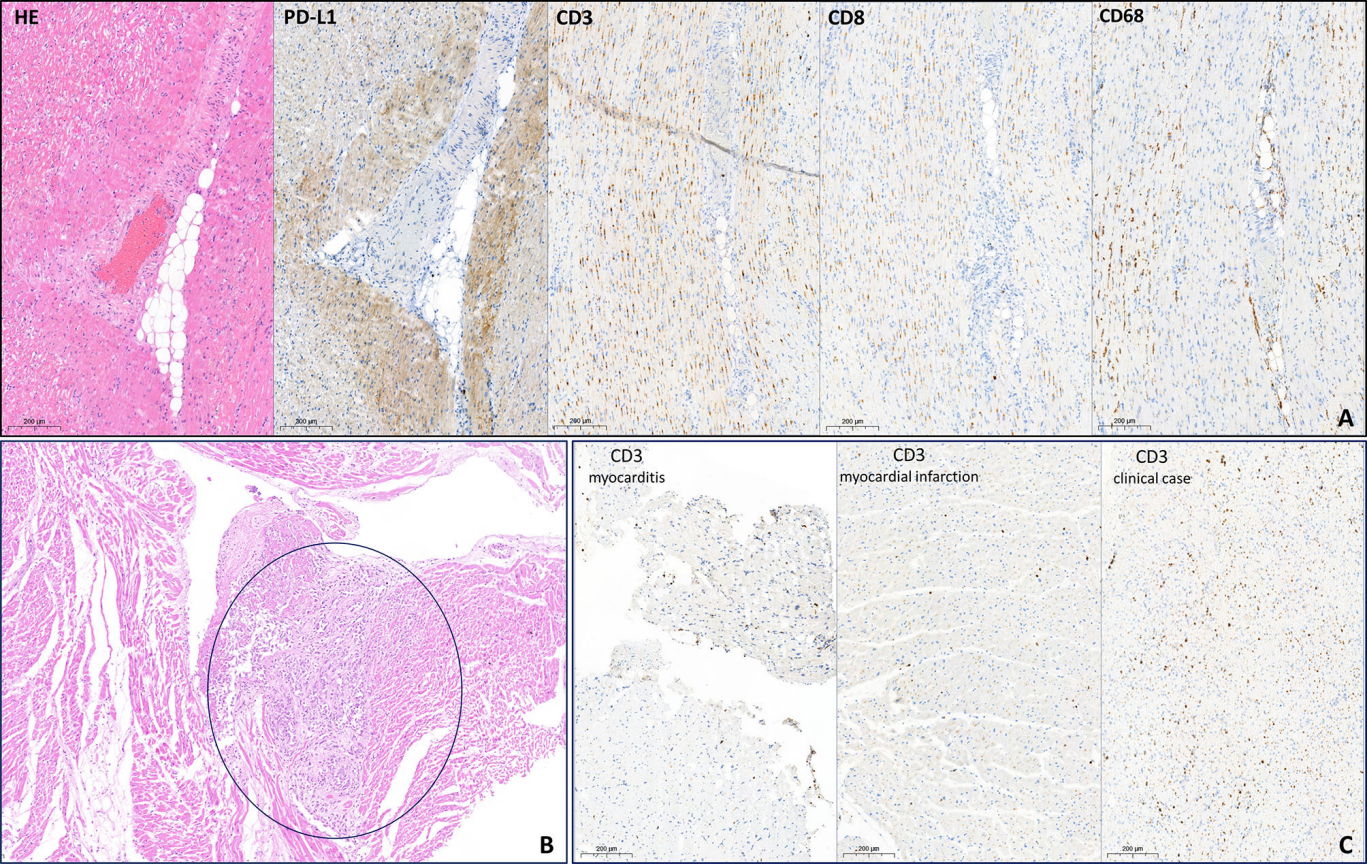
Histopathological evaluation of myocardium. **(A)** H&E and immunohistochemistry examination of myocardium free from metastasis. PD-L1 demonstrates cytoplasmic, membrane, and intravascular expression patterns. High infiltration of CD3 and CD68 cells and moderate infiltration of CD8 cells. **(B)** H&E. Subendocardial mucinous carcinoma micrometastases (circled in blue). **(C)** Comparison of CD3 expression in no-ICI-associated active lymphocytic myocarditis (>25 cells/mm^2^), in acute myocardial infarction (~15 cells/mm^2^), and in the patient after atezolizumab infusion (>50 cells/mm^2^). ICI, immune checkpoint inhibitors.

In coronary arteries (CAs), the lipid spots and plaques were presented with maximum diameter stenosis of 75%–80%. Also, multiple tumor emboli in CAs were detected. On the LV anterior wall, there was a small (2 cm) yellowish transmural focus with hemorrhages. Myocardium histological evaluation revealed cardiomyocyte necrosis with pronounced leukocyte infiltration. In addition to the standard autopsy protocol, we performed IHC examination with CD3, CD8, CD68, and PD-L1 (22C3 clone) antibodies. We showed high infiltration of CD3 and CD68 and moderate infiltration of CD8 cells. These results indicated the presence of both criteria to define current myocarditis: Dallas and IHC (>14 leukocytes/mm^2^, up to 4 monocytes/mm^2^ with CD3-positive T-lymphocytes >7 cells/mm^2^) (2). There was a diffuse expression of PD-L1 in the myocardium, which was more pronounced in perivascular zones. PD-L1 was presented with cytoplasmic, membrane, and intravascular expression patterns (**Figure 3A**).

Moreover, multiple mucinous carcinoma micrometastases were detected in the epicardium, including the supravalvular area (**Figure 3B**).

## Discussion

ICIs are a novel class of anticancer treatment with a wide range of indications and great clinical outcomes. These molecules inhibit immune checkpoints such as CTLA-4, PD-1, or PD-L1 on the surface of tumor and/or immune cells and activate an antitumor T-cell response. However, CTLA-4, PD-1, or PD-L1 could be expressed not only by tumor cells but also by the tumor microenvironment. In normal conditions and the context of different pathologies, these proteins play a protective role against autoimmune diseases through inhibition of the immune response. A high level of PD-L1 expression in human lungs with idiopathic pulmonary fibrosis (3) and an increase of PD-L1 expression in *Helicobacter pylori*-induced gastritis were shown (4). *In vitro* study revealed PD-L1 overexpression in damaged cardiomyocyte cell lines compared to intact cells (5). *In vivo* studies evidenced the cardioprotective role of the PD1/PD-L1 pathway against fulminant myocarditis manifestation (6, 7).

CV complications of ICI application in clinical practice are rare but associated with a very high mortality rate. Several studies indicated that ICI therapy may promote autoimmune myocarditis and increase the risk of ischemic CV disease progression (8, 9). However, data about potential risk factors for CV irAEs are very poor. A retrospective case–control study reported a comparison of ICI-related myocarditis, and other irAEs showed that preexisting CV risk factors and/or preexisting CV conditions were associated with the reporting of cases with ICI-associated myocarditis (10). Regrettably, there are no prospective studies that may support these data and define a group with an increased risk of irAEs.

We present a case of fulminant death after the first infusion of atezolizumab in the patient with preexisting HP and basal mid-range LV EF. In a retrospective study, Oren et al. showed that preexisting hypertension, heart failure, or stroke are associated with all-cause mortality. Also, they demonstrated that the frequency of checkpoint-associated myocarditis depends on the history of the acute coronary syndrome, heart failure, and age (>80 years) (11). So according to the patient’s known CVDs (HP and heart failure), his risk was 1.4%, but according to all CVDs (including those that were not diagnosed *in vivo*), his risk elevates to 2.8%.

The direct cause of death is not clear. According to pathomorphological evaluation, there are several possible reasons for cardiac dysfunction. Firstly, the multiple micrometastases in the myocardium and tumor emboli in CAs were revealed, which could partially explain the decrease of LV EF on par with the preexisting coronary atherosclerosis. Further, the area of acute MI in the left anterior descending artery supplying area was found. However, according to the size of transmural focus (2 cm), it is unlikely a leading and unique cause of sudden death. Lastly, the histological and IHC examination discovered pronounced inflammatory infiltration with high infiltration of CD3-, CD8-, and CD68-positive cells. Moreover, in comparable areas, high PD-L1 expression was detected. The patient was diagnosed with myocarditis as defined by Dallas and IHC criteria. Micrometastases were not considered as a possible reason for immune cells infiltration and high PD-L1 expression since IHC was performed on samples free from tumor cells. To exclude an acute MI as a possible reason for high inflammatory infiltration, we compared CD3 expression between the present patient sample, endomyocardial biopsy sample with active lymphocytic myocarditis (not ICI-associated), and myocardium sample of a patient who died in an acute period of MI (**Figure 3С**). An extremely high level of inflammatory cells was observed only in the present patient sample, but not in usual MI. These results imply that the observed pattern could not be a consequence of acute myocardial ischemia only.

On the other hand, a high level of PD-L1 expression may be a preexisting cardioprotective mechanism caused by cardiac damage with metastasis, tumor emboli, and hemodynamically significant coronary stenosis. Our recent study showed an increase in PD-L1 expression in the myocardium of patients with ischemic heart disease and dilated cardiomyopathy compared to the control group without any cardiovascular diseases (12). Hence, we hypothesize that the infusion of anti-PD-1 ICI blocked the PD-1/PD-L1 pathway, which resulted in massive inflammatory activation with severe heart damage.

We demonstrated heart damage of multiple causes that was difficult to diagnose during a patient’s lifetime. This case actualizes the problem of absent data about potential high-risk groups for CV irAEs and the gap in knowledge of the contribution of previous CV diseases.

## Data Availability Statement

The original contributions presented in the study are included in the article/supplementary material. Further inquiries can be directed to the corresponding author.

## Ethics Statement

Written informed consent was obtained from the individual(s) for the publication of any potentially identifiable images or data included in this article.

## Author Contributions

EK and MSi: preparing manuscript. MSt, EA, and TS: patient management, providing diagnostic, and treatment results. VK: histopathological examination. FM and OM: manuscript revision. All authors listed have made a substantial, direct, and intellectual contribution to the work and approved it for publication.

## Funding

The study has been supported by a grant from the Ministry of Science and Higher Education of the Russian Federation (agreement no. 075-15-2020-901).

## Conflict of Interest

The authors declare that the research was conducted in the absence of any commercial or financial relationships that could be construed as a potential conflict of interest.

## Publisher’s Note

All claims expressed in this article are solely those of the authors and do not necessarily represent those of their affiliated organizations, or those of the publisher, the editors and the reviewers. Any product that may be evaluated in this article, or claim that may be made by its manufacturer, is not guaranteed or endorsed by the publisher.
